# Female Sexual Function During the Menopausal Transition in a Group of Iranian Women 

**Published:** 2016-06

**Authors:** Tahereh Eftekhar, Mahboobeh Dashti, Mamak Shariat, Fedyeh Haghollahi, Firoozeh Raisi, Akram Ghahghaei-Nezamabadi

**Affiliations:** 1 Vali-e-Asr Reproductive Health Research Center, Tehran University of Medical Sciences, Tehran, Iran; 2 Department of Obstetrics and Gynecology, Vali-e-Asr Hospital, Tehran University of Medical Sciences, Tehran, Iran; 3 Maternal-Fetal and Neonatal Health Research Center, Tehran University of Medical Sciences, Tehran, Iran; 4 Psychiatric and Psychological Research Center, Roozbeh Hospital, Tehran University of Medical Sciences, Tehran, Iran

**Keywords:** Sexual Dysfunction, Menopause, Psychological Sexual Dysfunction, Libido, Arousal, Female Sexual Function Index

## Abstract

**Objective:** To determine the prevalence of sexual problems in Iranian women and association of sexual dysfunction with menopausal symptoms.

**Materials and methods:** In this cross-sectional study, 151 married women with the age of 40-60 yearsold who were referred for treatmentto Department of Gynecology in Vali-e-Asr Hospital (Tehran, Iran) from April to July 2012, were recruited. They were evaluated concerning their sexual function in the domains of desire, arousal, lubrication, orgasm, satisfaction and pain with the female sexual function index (FSFI) questionnaire.Menopause rating scale (MRS) was developed for the diagnosis and quantification of climacteric symptoms.

**Results:** Total frequency of sexual dysfunction was 53% with the domains of lubrication, arusal and desire being commonly affected 62%, 70% and 98.5% of cases respectively. There is a relationship between severity of somatic and urogenital symptoms with sexual dysfunction (p = 0.03, p = 0.00 respectively).

**Conclusion:** A considerable percentage of women experienced sexual dysfunctions in this period. Somatic and urogenital symptoms during the menopausal period could be a factor to maintain or intensity of sexual dysfunctions.

## Introduction

Menopause is a transitional and physiological event in a woman's life that occurs in all women who reach midlife (1).

The frequency of ovulation decreases by the age of 40 and reproductive function ceases within the following 15 years that is due to the cessation of ovarian activity and subsequent hormonal changes (especially in the absence of estrogen. Several problems are associated with symptoms, which can affect the quality of life in women (2-4).

The menopausal transition is a turning point for many womenand is associated with physiologicaland psychological changes that influence sexuality. Physiological changeis because of decline in circulating estrogen levels. Estrogen deficiency initially is related to irregular menstruation and can diminish vaginal lubrication. Persistent estrogen loss is associated with changes in the vascular, muscular, and urogenital systems, and also alterations in mood, sleep, and cognitive function. These changes influence sexual function both directly and indirectlyand cause sexualproblems (6, 7).

Estrogen deficiency is associated with symptoms like hot flushes, night sweats, insomnia and vaginal dryness (7). 

Sexual problem, or sexual dysfunction, is a problem during any phase of the sexual response cycle and resulting from physical, social, and psychological factors that prevent experiencing the individual or couple satisfaction from the sexual activity (8). Epidemiological studies in the United States have estimated that female sexual dysfunction (FSD) affected 43% of women in the general population over the past 12 months (9). 

In the United Kingdom, 5.8% of women have reported recent sexual dysfunction, and 15.5% have reported lifelong sexual dysfunction (8).

 The rate of FSDfor middle aged women in Latin America is approximately 58% (10). In a survey conducted in six European countries on 1805 post-menopausal women (age range: 50–60 years), one-third (34%) reported that they experienced a reduced sex drive, whereas half (53%) noticed that they had become less interested in sex (11).

Frequency of sexual dysfunctions in the reproductive periodin Iran was 38% and in the menopause and late menopausal periods was 72.4% (12, 13).

The results on prevalence differ across studies depending on several factors such as sample size, design, menopausal status and country (14).With increasing life expectancy, it is necessary to be done studies about the middle-aged women's sexual health.

Therefore, the aim of this study was to assess the prevalence of sexual dysfunction and association of sexual dysfunction with menopausal symptoms on Iranian married women with the age of 40-60 years.

## Materials and methods

This cross-sectional study was conducted in the health practitioner at Department of Gynecology in Vali-e-Asr Hospital (Tehran, Iran) for two months in 2012. A total of 250 married women with the age 40-60 years, were recruited after obtaining a written informed consent.This study was confirmed by the Ethics Review Committee of Vali-e-Asr Hospital (Tehran, Iran). The following participants were excluded: those who were under treatment for sexual disorders; those who had used hormones up to 60 days before the selection process; over the past two monthsstay in the hospital, any medical or psychiatric condition requiring medication at present, any past history of diagnosed mental illness or specific medical diseases among couples, women whose spouses had premature ejaculation or sexual disabilities. The questionnaire response rate was 60%. Our final sample size was 151. In this study three questionnaires including Demographic, Menopause rating scale (MRS) and Female Sexual Function Index (FSFI) were adjusted.

Sexual function was assessed by using the FSFI questionnaire in Persian which was previously translated and validated (13). Sexual dysfunction was assessed by using Female Sexual Function Index (FSFI) scale (15-17).

The scale is a 19-items questionnaire, developed as multidimensional self-report instrument for the assessment of the key dimensions of sexual function in women in last 1 month.

This questionnaire consists of questions in 6 domains including desire, arousal, lubrication, orgasm, satisfaction and pain. The items of each scale are divided into 6-domains which include desire (2 questions), subjective arousal (4 questions), lubrication (4 questions), orgasm (3 questions), satisfaction (3 questions) and pain (3 questions). 

Since in the FSFI questionnaire, the questions are not equal to each other, initially for the equiponderate the fields together, scores of questions in each area are added together and then multiplied by the number of factors. For questions on sexual desire domain scores considered (1-5), orgasm, lubrication, pain (0-5) and sexual satisfaction (5 or 0, 1). A score of zero indicates that during the past 4 weeks, she has not had sexual activity.

Based on weight of the domains, the maximum score for each area of 6 and 36 will be for the total scale. The cut off score are to the field of sexual desire (3.3), sexual orgasm (3.4), lubrication (3.7), pain and satisfaction (3.8) and for the total scale, the minimum score is equal to 28. Accordingly scores less than mentioned cases, the disorder is considered.

The total FSFI score is the sum of all scores obtained in each 6domain was 36 .The higher score meansthe better the sexuality.The scoreslowerthan 26.55 was considered as the cut-off value for diagnosis of female sexual dysfunction (18).

Socio-demographic and clinical data were collected using a specially designed questionnaire. 

The Menopause Rating Scale (MRS) is a self-assessment process for the diagnosis and quantification of climacteric symptoms. MRS was developed, standardized and validated in the 1990s by a group of experts (19, 20).

Menopause rating scale questionnaire (MRS) was assessed by the Persian translators. It was assessed by 5 experts opinion in Sexology on15 women within twoweeks. The validity and reliabilitywas confirmed by pre and posttest (Cronbach’s alpha = 0.816).

MRS is the scoring by point with increasing severity of subjectively perceived complaints in each of the 11 items (severity expressed in 0-4 points in each item).

The minimal/maximal scores vary between the three dimensions depending on the number of complaints allocated to the respective dimension of symptoms:

Psychological symptoms: 0 to 16 scoring points (4 symptoms: depressed, irritable, anxious, exhausted); somato-vegetative symptoms: 0 to 16 points (4 symptoms: sweating/flush, cardiac complaints, sleeping disorders, joint & muscle complaints); Urogenital symptoms: 0 to 12 points (3 symptoms: sexual problems, urinary complaints, vaginal dryness). Each item is accompanied by with 5-point Likert scale were measured. The options of never, and very severe were given 5 score.

Total MSR score was 11-55. However if overall score rating for each domain was less; fewer menopausal symptoms were experienced. For participants both questionnaires were completed by face-to face interviewers. They were asked to report any symptoms and severity was listed in the MRS questionnaire during the last month.

Data were analyzed with SPSS software version 17 (SPSS Inc. Chicago, IL, USA), P-value of 0.05and lower was considered significant. Data are expressed as mean, standard deviations (SD) and percentages.

Mean and SD were used to evaluate descriptive data. χ^2^ Test was used to compare categorical variables, and Student t- test and analysis of variance were used to compare the continuous variables (FSFI variable).

Bivariate correlations were investigated by Pearson product-moment correlation coefficient. Cronbach’s alpha coefficient was calculated to evaluate reliability of questionnaire and it was 0.816.

## Results

Froma total of151women 40-60 years participated in this study during the menopausal transition, 53% had and 47% did not have impaired sexual function.The majority of cases 106 patients (70%) were under 50 years of age, menopause and their level of education was high school or less (Table 1).

**Table 1:** Participant characteristics

**Variable**	**n (%)**
Age group (n/%)	
< 50 y	106 (70%)
> 50 y	45 (30%)
Education (n/%)	
< Diploma	59 (39%)
Diploma	62 (41%)
> Diploma	30 (20%)
Marriage duration n (%)	
< 10	1 (1%)
10-20	42 (28%)
> 20	108 (72%)
Delivery no n (%)	
2-3	95 (63%)
> 3	5 (37%)
BMI (mean ± SD)	28.49 ± 4.93
Cigarrete user n (%)	
No	80 (96%)
Yes	4 (4%)
Surgery history n (%)	
No	145 (96%)
Yes	6 (4%)
Menopause n (%)	
No	106 (70%)
Yes	45 (30%)

We also compared the participants who reported FSD with those who did not report such problems.

We found that the mostfrequentdisorderswere in lubrication, arousal anddesire (p < 0.05) (Table 2). Also, subjects with sexual dysfunction in these three dimensions are more impaired.

**Table 2:** Sexual function domains in subjects

**Sexual function **	**Normal** **n (%)**	**Dysfunction** **n (%)**	**p value**
**Variable**			
Desire	57 (38%)	94 (62%)	0.003
Arousal	45 (30%)	106 (70%)	0.000
Lubrication	45 (30%)	106 (70%)	0.000
Orgasm	77 (51%)	74 (49%)	0.874
Satisfaction	85 (56%)	66 (44%)	0.143
Pain	72 (48%)	79 (52%)	0.626
Total Sexual function	71(47%)	80 (53%)	0.515

The highest frequency of somatic, psychological and urogenital symptoms were inmild and moderatelevels. (95%, 92% and 97% respectively) (p < 0.000) (Table 3).

**Table 3:** The frequency of the menopause Rating Scale (MRS) in the cases of study

**Domain**	**Mild** **n (%)**	**Moderate** **n (%)**	**Sever** **n (%)**	**p-value**
**Groups**				
Somatic	114 (75%)	30 (20%)	7 (5%)	0.000
Psychologic	64 (42%)	76 (50%)	11(8%)	0.000
Urogenital	102 (67%)	45 (30%)	4 (3%)	0.000

By using the Chi-square test, there is a relationship between severity of somatic and urogenital symptoms withsexual dysfunction. This means that seven women with severe somaticsymptoms and also four women withsever urogenital symptoms have sexual dysfunction (Table 4). While in women with normal sexual function, no sever symptoms in the somatic and urogenital dimension were observed.

**Table 4:** Relationship between sexual function and MRS (Menopause rating scale) in subjects

**Sexual function** **MRS Domain**	**Normal** **n (%)**	**Dysfunction** **n (%)**	**p-value**
Somatic			0.03
Mild	57 (37%)	57 (38%)	
Moderate	14 (9%)	16 (11%)	
Sever	0	7 (5%)	
Psychologic			0.14
Mild	36 (24%)	28 (18%)	
Moderate	31 (20%)	45 (30%)	
Sever	4 (3%)	7 (5%)	
Urogenital			0.00
Mild	59 (39%)	43 (29%)	
Moderate	12 (8%)0	33 (22%)	
Sever	0	4 (3%)	

There was no relationship between severity of psychological factors and sexual dysfunction.

T-test analysis between the two groups of menopause and non-menopause women showed thatthey are differentin the dimensions of sexual dysfunction, and significant differences were observed between the two study groups. This means that in menopausal women, sexual dysfunctionis higher than non-menopausal women (Table5).

**Table 5:** Relationship between FSFI (female sexual function index) and MRS (Menopause rating scale) in menopausal and non-menopausal women.

**Group**	**Menopause** **mean ± SD**	**Non-menopause** **mean ± SD**	**p-value**
**Variable**			
FSFI			
Desire	2.1 ± 1.06	3.2 ± 1.2	0.001
Arousal	1.2 ± 1.4	2.98 ± 1.7	0.000
Lubrication	1.5 ± 1.8	3.2 ± 1.8	0.001
Orgasm	1.3 ± 1.8	3.5 ± 1.9	0.000
Satisfaction	1.9 ± 2.2	3.9 ± 2.1	0.000
dysparunia	2.5 ± 2.9	3.8 ± 2.2	0.40
MRS			
Somatic	3.6 ± 3.3	4.4 ± 2.8	0.75
Psychology	5.2 ± 3.5	6.17 ± 3.5	0.85
urogenital	2.2 ± 2.18	4.2 ± 2.7	0.08

But in each of the three dimensions of menopausal symptoms we did not find significant difference in the average scoring. This symptomsin menopausal and non-menopausal women were almost identical (Table5).

## Discussion

This study was aimed to assess the prevalence of sexual dysfunction and association of sexual dysfunction with menopausal symptoms on 151 women aged 40-60 years during the menopausal transition. The results of the present study showed that 53% of the women in this study are suffering from sexual dysfunction.

The rate of FSD for middle aged women in Latin America is approximately 58% (10).

In Indian women FSFI total scores suggested FSD in two-thirds of the 149 women (73.2%) (22).

In Iran we showed that the relative frequency of sexual dysfunctions was 38% in the reproductive period and 72.4% in the menopause and late menopausal periods (12, 13) 

In Nigeria the incidence of these dysfunctions was reported 40.4%, (23) and in Brazilia was reported 35%. Overall sexual dysfunction was evaluated in women with or without a sexual partner (24).

In Latin America the reported sexual dysfunction in middle aged womenwas 55.7%, (25).

Sexual dysfunction in Brazilian women during the menopausal transitional periods was reported 57.4% that consistent with our study (26).

The difference between the obtained results in our country and other countries can be due to differentage groups and differences in racial, religious and cultural aspects, sample size, attitudes of these women toward the menopause phenomenon and the study inclusion criteria. In this study, only the married women aged 40-60 was considered.

In our study the frequency of sexual functions based on normal and dysfunction that dysfunction in arousal (70%), lubrication (70%) and desire (62%) were reported.

Also In normal Korean womenwith the age of 18-52 years, problems of desire (44.0%), arousal (49.0%), lubrication (37.0%), orgasm (32.0%), satisfaction (37.0%), and pain (34.6%) were detected (22).

Lack of sexual desire by 26.7%; pain during sexual intercourse by 23.1% and orgasmic dysfunction by 21%in Women aged over 40 y were reported in Abdo's study. Also, multiple analysis showed no association between menopausal status and sexual dysfunction (26).

The distributions of these disorders in Iran during the menopausal period were as Follows: arousal disorder (75.3%), sexual desire disorder (62.6%), orgasmic disorder (56.3%), dyspareunia (34.9%) (12).

In an another study in Iran , the prevalence of sexual dysfunction in women aged 20-60 years old was 39% in those > 50 years, 37% reported orgasmic disorders, 35% desire disorders and 30% arousal disorders (18).

FSFI domain scores suggested difficulties with desire in 77.2%; arousal in 91.3%; lubrication in 96.6%; orgasm in 86.6%, satisfaction in 81.2%, and pain in 64.4% age above 40 years Indian women (21).

The longest-duration population-based study, the Melbourne Women’s Midlife Health Project, found a significant decrease inwomen’s desire, arousal, orgasm and frequency of sexual activity, with a rate of sexual dysfunction that ranged from 42% to 88% throughout the menopausal transition (27, 28).

It is estimated that 40 to 45% of adult women suffer from some form of sexual dysfunction (29). Desire and orgasmic dysfunction are the most frequently reported problems. A systematic review of prevalence rates have found a mean rate of 64% for desire problems; 35% for orgasmic difficulties; arousal problems, 31%; and for pain, 26% (30).

The considerable differencesare between the obtained results of sexual function domains in our country and other countries (11,21,22,13,23,29) that can be due to aging, changes in libido, body shape, number of sexual intercourses, low physical activity, health of a person,unhappy marriage and relationship factors in sexual function during the natural menopausal transition.Thus it is needed for more comprehensive research in this age group (18,31,32).

These rates defend the interest that has been paid to sexual function recently. Also, the prevalence of female sexual dysfunction in populations from various countries, cultures, and age groups effect on women's lives today. Therefore, these findings suggest future directions for the delivery of services addressing the prevention and treatment of FSD.

The results of this study showed that the prevalence of sexual dysfunction in menopausal women is higher than non-menopausal women. Female sexual function declines with the natural menopausal transition (33).

The progressive decline of sexual hormones interacts with the aging process and many psychosocial stressors modulate for sexual symptoms (low sexual desire, poor arousal and lubrication, dyspareunia, orgasmic dysfunction and lack ofsatisfaction) (34).

The results of our study showed that in women with sever somatic and urogenital symptoms, sexual dysfunction was more common. It seems that in this age group, severity of symptoms can cause sexual dysfunction. The result of the present study is consistent with the study of Senturk Erenel that women have different levels of menopausal symptoms, and the severity of menopausal symptoms increases sexual dysfunction (35).

Since, in this study allsamples are in the menopausal transition periods, therefore menopausal and non-menopausal women were not different in means of somatic, psychology and urogenital symptoms.

Women in the menopausal transition period are affected with menopausal symptoms (36).

These findings have great implications for sexual dysfunction prevention and perhaps symptomatic treatment will lead to improved sexual dysfunction and often affected their quality of life (QoL) (37).

One of the main limitations of our study was that the participants were selected in a clinical setting, and the results were not necessarily representative of the general population of Iran. Lack of variables affecting sexual function are the other limitations of this study and more detailed studies are necessary.

Nevertheless, the findings of the current study are relevant to clinical practice and the complex symptomatology in women attending a clinic.

Talk about sexual problems is an essential part of healthcare, and early recognition of those menopausal women who are distressed by FSD is a critical step in effective therapeutic management (38).

## Conclusion

As a result, our research shows a significant proportion of women in this age, experience sexual dysfunction and severity of menopausal symptoms in the somatic and urogenital dimensions are associated with the creation or enhancement of sexual dysfunction. It is important to recognize sexual concerns well-timed so as to found appropriate symptomatic medical treatment.

So, sexual disorders, by treating symptoms at this age, maybe reduced.

Because of high percentage of sexual desire and arousal disorder during the menopausal transition, therefore sexual activity and satisfaction is one of the most important aspect of human's life which is largely ignored and it seems we need more investigations, especially in developing countries.

Also, we should have emphasis on counseling and education about sexual activities during the menopausal period.
